# Relationship Among Inflammation, Overweight Status, and Cognitive Impairment in a Community-Based Population of Chinese Adults

**DOI:** 10.3389/fneur.2020.594786

**Published:** 2020-12-08

**Authors:** Jing Wang, Anxin Wang, Xingquan Zhao

**Affiliations:** ^1^Department of Neurology, Beijing Tiantan Hospital, Capital Medical University, Beijing, China; ^2^China National Clinical Research Center for Neurological Diseases, Beijing, China; ^3^Center of Stroke, Beijing Institute for Brain Disorders, Beijing, China; ^4^Beijing Key Laboratory of Translational Medicine for Cerebrovascular Disease, Beijing, China; ^5^Department of Epidemiology and Health Statistics, School of Public Health, Capital Medical University, Beijing, China; ^6^Beijing Municipal Key Laboratory of Clinical Epidemiology, Beijing, China

**Keywords:** high-sensitivity C-reactive protein (hs-CRP), cognitive impairment, overweight, body mass index, inflammation

## Abstract

**Purpose:** To determine the association between overweight and high-sensitivity C-reactive protein (hs-CRP) with the odds of cognitive impairment as well as its subtypes based on the Asymptomatic Polyvascular Abnormalities Community (APAC) study in China.

**Materials and methods:** We conducted a cross-sectional analysis of the follow-up data of 2012 from the APAC study. The Chinese version of the MMSE was used as a cognitive screener, and an MMSE score <24 is generally accepted as indicating cognitive impairment. Multivariable logistic regression was used to estimate the interactions of hs-CRP levels with body mass index (BMI) on the effects of cognitive impairment and its subtypes.

**Results:** Three thousand eight hundred seventy-five participants aged 40–90 years (median age 51.64 y) were enrolled in this study, and 1,788 (46.1%) were overweight. Before and after adjusting for confounders, such as age, sex, BMI, education, current smoking, drinking, physical activity, hypertension, hyperlipidemia, diabetes, and hs-CRP, elevated hs-CRP levels were associated with cognitive impairment in normal-weight participants (crude OR: 2.08, 95%CI: 1.28–3.37, *p* = 0.003; adjusted OR: 2.06, 95%CI: 1.03–4.10, *p* = 0.04), but not in overweight participants. There was no statistically significant evidence for the interaction between hs-CRP and BMI on any cognitive sub-item.

**Conclusion:** Elevated hs-CRP levels increase the odds of cognitive impairment in normal-weight participants, but not in overweight participants.

## Introduction

Cognitive impairment has become an obvious and important public health problem among elderly patients, especially with the increasing trend in longevity of these subjects. Nearly 10–45% of elderly patients experience some degree of cognitive impairment (1), resulting in increased mortality (2) as well as social and economic burden (3). This trend has been projected to be more dramatic by 2050, and approximately half of the global distribution of incident cognitive impairment will be in Asia (4).

Previous studies and some meta-analyses have demonstrated that being overweight and obese are harmful to cognition (5–10), while some studies showed that being overweight maybe helpful to cognition (11, 12). Deckers et al. found that the association between being overweight and the rate of cognitive decline was definitely affected by age (13). Some meta-analysis showed that being overweight was positively associated with dementia in middle-aged people, but negatively associated with cognitive impairment in middle- and old-aged people (5). However, another meta-analysis showed that weight loss in overweight subjects will improve cognition (14), which would indirectly reflect the association between being overweight and cognitive impairment. Inflammation is the body's defense against harmful stimuli, and accumulated evidence has shown that obesity can result in systemic and central inflammation and lead to cognitive impairment (15). High-sensitivity C-reactive protein (hs-CRP) is an indicator of inflammation and plays an important role on overweight-associated cognitive impairment. Body mass index (BMI) has been recognized as the most precise value in the evaluation and classification of body weight.

Because studies on the association of BMI, hs-CRP, and the odds of cognitive impairment (including its subtypes) are particularly limited, we aimed to further explore these associations based on a community study in China.

## Methods

### Study Design and Subjects

In the present investigation, we conducted a cross-sectional analysis of the follow-up data of 2012 from the Asymptomatic Polyvascular Abnormalities Community (APAC) study. The APAC is a community-based, prospective, long-term, follow-up observational study on the epidemiology of asymptomatic polyvascular abnormalities in Chinese adults. It is part of the Kailuan study, which has been described previously (16). A sample of 7,000 subjects, ≥40 years of age, was randomly selected from the Kailuan cohort, using an age- and sex-based stratified random sampling, and was based on the data of state census in 2010. The sample size was calculated according to detection of 7% of event rate with 0.7% precision (α = 0.05). The response rate was assumed to be >80%. An initial cohort of 5,852 subjects participated in the study, and 5,816 completed the baseline survey and assessment from June 2010 to June 2011. Among the 5,816 individuals, 376 were excluded for the following reasons: (1) history of stroke, transient ischemic attack, and coronary disease at baseline; and (2) presence of neurologic deficits which were estimated by experienced doctors. A total of 5,440 participants were enrolled in the APAC study. During the baseline survey, all participants underwent a questionnaire-based assessment and clinical and laboratory examinations. Follow-up data was collected in 2012. We further excluded 1,565 participants who had an incomplete data of Mini-Mental State Examination (MMSE) score, leaving 2,203 men and 1,672 women in the final analyses. The APAC study was performed according to the tenets of the Helsinki Declaration and was approved by the Ethics Committees of the Kailuan General Hospital and Beijing Tiantan Hospital. Written informed consent was obtained from all participants and approved by the above ethics committees.

### Measurement of Indicators

A questionnaire was used to obtain baseline information such as age; sex; smoking status; and medical history including hypertension, diabetes, hyperlipidemia, and medications prescribed. Weight, height, and blood pressure (BP) were measured during the baseline interview, and BMI was calculated using the formula: body weight (kg) divided by the square of height (m^2^). BP was the average of two readings at rest. If the two measurements differed by more than 5 mm Hg, then an additional reading was taken, and the average of the three readings was used. We further categorized the subjects according to different parameters, i.e., age (<65 years and ≥65 years), BMI [normal weight (<25 kg/m^2^) and overweight (≥25 kg/m^2^)], and smoking status (current smoker who smokes at least one cigarette per day and non-smoker). Drinking was defined as consumption of ≥100 mL/day of any alcohol-containing liquid for more than 1 year. The education level was categorized as “illiterate or primary school,” “middle school,” or “high school or higher.”

Blood samples were obtained after an overnight fast in EDTA tubes at the baseline interview. Fasting blood glucose was measured with the hexokinase/glucose-6-phosphate dehydrogenase method. Cholesterol and triglyceride were measured enzymatically (Mind Bioengineering Co. Ltd, Shanghai, China). High-sensitivity (hs)-CRP was measured by high-sensitivity nephelometry assay (Cias Latex CRP-H, Kanto Chemical Co. Inc, Tokyo, Japan). All blood variables were measured using an auto-analyzer (Hitachi 747; Hitachi, Tokyo, Japan) at the central laboratory of Kailuan General Hospital.

### Assessment of Cognitive Function

The Chinese version of the MMSE, which was first translated and modified by Katzman et al. (17), was used as a cognitive screener. The score range was from 0 to 30 points and further divided into five sub-scores that assessed orientation, registration, attention and calculation, recall, and language, with a lower score reflecting greater cognitive impairment. Specially trained physicians or assistants tested participants face-to-face with an MMSE in 2012. An MMSE score <24 is generally accepted as indicating cognitive impairment (18, 19). Specificity was found to be between 80 and 100% (20). The score of each MMSE sub-item was classified into normal/impaired group according to the third quartile of each score.

### Definition of Hypertension, Diabetes, and Hyperlipidemia

Information on demographic variables (e.g., age and sex) and smoking status, alcohol intake, and medical history was collected via questionnaires administered by the research doctors at the baseline interview. Hypertension was defined as the presence of any of the following: a history of hypertension, using antihypertensive drugs, systolic blood pressure ≥140 mmHg, or diastolic pressure ≥90 mmHg. Diabetes mellitus was diagnosed as the presence of any of the following: a history of diabetes mellitus, currently treated with insulin or oral hypoglycemic agents, or fasting blood glucose level ≥7.0 mmol/L. Hyperlipidemia was defined as the presence of any of the following: a history of hyperlipidemia, current use of cholesterol-lowering agents, or total cholesterol level ≥5.17 mmol/L or triglycerides ≥1.7 mmol/L.

### Classification of Hs-CRP

According to the guideline from the Centers for Disease Control and Prevention and the American Heart Association, hs-CRP levels were categorized into two groups: low–medium risk (<3 mg/L) and high risk (≥3 mg/L) (21).

### Statistical Analysis

Statistical analyses were performed using the SAS software, version 9.3 (SAS Institute, Cary, North Carolina, USA). All participants were classified into two subgroups according to BMI. In each subgroup, baseline characteristics were compared according to hs-CRP levels. As all the continuous variables showed skewed distribution, median with interquartile range (IQR) was used for analysis, and the Wilcoxon rank sum tests were used for comparison. Categorical variables were presented as frequencies (percentage) and compared using the chi-square tests or Fisher's exact test as appropriate.

The interactions of BMI on the effects of cognitive impairment as well as its subtypes were investigated with the use of crude and multivariable logistic regression. In the multivariable model, demographic variables such as age, sex, BMI, education, current smoking, drinking, physical activity, hypertension, hyperlipidemia, diabetes, and hs-CRP were adjusted. Odds ratios (ORs) with a 95% confidence intervals (CIs) were reported. As the *P*-values for interaction were all >0.05, no interaction term was added into the main logistic model. Additionally, the relationship between BMI with hs-CRP stratification and cognitive impairment was further analyzed in total and subgroups, such as age, sex, and education. All statistical analyses were two-tailed, and a *P*-value of 0.05 was considered to indicate as statistically significant.

## Results

### Baseline Characteristics

Of the 5,440 participants recruited to the APAC study, 3,875 subjects (1,672 women and 2,203 men) were included in the final analysis. No baseline variable of interest was missing overall. The baseline characteristics of patients included and those not included in this analysis were basically similar, except that the included participants were younger, mostly women, had more access to higher education, had a history of hyperlipidemia, and showed lower systolic blood pressure (SBP), FPG, and hs-CRP levels at baseline (Table 1).

**Table 1:** Baseline characteristics of participants included vs. not included in this study.

**Characteristics**	**Patients included**	**All other patients**	***P*-value**
*N*, %	3,875 (71.2)	1,565 (28.8)	
Age (median, years)	51.64 (45.32, 59.62)	54.78 (46.67, 68.64)	<0.01
BMI (median, kg/m^2^)	24.74 (22.67, 27.01)	24.74 (22.64, 27.18)	0.58
Female *n*(%)	1,672 (43.15)	511 (32.65)	<0.01
Education (*n*%)			<0.01
Illiteracy or primary school	404 (10.43)	259 (16.55)	
Middle School	1,671 (43.12)	723 (46.20)	
High school or higher	1,800 (46.45)	583 (37.25)	
Smoking status (*n*%)	1,236 (31.90)	501 (32.01)	0.95
Drinking (*n*%)	554 (14.30)	222 (14.19)	0.93
**Medical history (*****n*****%)**			
Hypertension (*n*%)	968 (24.98)	425 (27.16)	0.10
Diabetes (*n*%)	289 (7.46)	135 (8.63)	0.15
Hyperlipidemia (*n*%)	478 (12.34)	138 (8.82)	0.0002
Lipid-lowering drugs (*n*%)	61 (1.57)	14 (0.89)	0.05
SBP (median, mmHg)	130.00 (118.67, 140.00)	130.00 (120.00, 146.00)	<0.01
LDL (median, mmol/L)	2.60 (2.16, 3.04)	2.61 (2.18, 3.09)	0.33
HDL (median, mmol/L)	1.57 (1.31, 1.89)	1.59 (1.30, 1.91)	0.66
TG (median, mmol/L)	1.30 (0.93, 1.91)	1.32 (0.95, 1.96)	0.56
TC (median, mmol/L)	4.95 (4.38, 5.62)	4.92 (4.32, 5.66)	0.42
FPG (median, mmol/L)	5.19 (4.81, 5.75)	5.30 (4.88, 5.92)	<0.01
Hs-CRP (mg/L)	0.97 (0.50, 2.10)	1.10 (0.60, 2.40)	<0.01

*BMI, body mass index; FPG, fasting plasma glucose; HDL, high-density lipoprotein cholesterol; hs-CRP, high-sensitivity C reactive protein; LDL, low-density lipoprotein cholesterol; SBP, systolic blood pressure; TC, total cholesterol; TG, triglycerides*.

The baseline characteristics of all participants are shown in Table 2. Three thousand eight hundred seventy-five (3,875) participants aged 40–90 years (median age 51.64 y) were enrolled in this study, and 1,788 (46.1%) were overweight. Participants with elevated levels of hs-CRP were older, weighed more, and had a higher incidence of prior hypertension, diabetes, hyperlipidemia, and stroke. The proportion of participants with elevated hs-CRP levels was higher in the overweight group than in the normal weight group (22.99 vs. 13.08%, *p* < 0.01). The median hs-CRP in the overweight and normal groups were 1.30 and 0.80, respectively (*p* < 0.01). In the overweight group, participants with elevated levels of hs-CRP were older, weighed more, more likely to engage in drinking, and this group had a higher incidence of prior hypertension, diabetes, and hyperlipidemia. In the normal weight group, participants with elevated levels of hs-CRP were older, weighed more, performed more physical activity, and had a higher incidence of prior hypertension and hyperlipidemia.

**Table 2:** Baseline characteristics of participants stratified by hs-CRP levels.

**Characteristics**	**Hs-CRP < 3 mg/L**	**Hs-CRP ≥ 3 mg/L**	***P-value***	**Hs-CRP < 3 mg/L, BMI < 25 kg/m^**2**^**	**Hs-CRP ≥ 3 mg/L, BMI < 25 kg/m^**2**^**	***P-value***	**Hs-CRP < 3 mg/L, BMI ≥ 25 kg/m^**2**^**	**Hs-CRP ≥ 3 mg/L, BMI ≥ 25 kg/m^**2**^**	***P-value***
Number, %	3,191 (82.35)	684 (17.65)		1,814 (86.92)	273 (13.08)		1,377 (77.01)	411 (22.99)	
Age (median, years)	51.18 (45.07, 58.99)	53.84 (46.97, 63.74)	<0.001	50.93 (45.00, 58.94)	54.03 (47.07, 66.51)	<0.001	51.49 (45.17, 59.00)	53.78 (46.83, 61.94)	<0.001
BMI (median, kg/m^2^)	24.46 (22.49, 26.58)	26.03 (23.67, 28.04)	<0.001	22.82 (21.48, 23.94)	23.31 (22.04, 24.21)	0.0006	27.04 (25.95, 28.58)	27.68 (26.49, 29.38)	<0.001
Female *n*(%)	1387 (43.47)	285 (41.67)	0.40	880 (48.51)	117 (42.86)	0.09	507 (36.82)	168 (40.88)	0.15
Education *n*(%)			0.02			0.22			0.09
Illiteracy or primary school	312 (9.78)	92 (13.45)		163 (8.99)	31 (11.36)		149 (10.82)	61 (14.84)	
Middle School	1,387 (43.47)	284 (41.52)		755 (41.62)	101 (37.00)		632 (45.90)	183 (44.53)	
High school or higher	1,492 (46.76)	308 (45.03)		896 (49.39)	141 (51.65)		596 (43.28)	167 (40.63)	
Smoking status (*n*%)	1,014 (31.78)	222 (32.46)	0.75	563 (31.04)	78 (28.57)	0.44	451 (25.22)	144 (8.05)	0.40
Drinking (*n*%)	460 (14.42)	94 (13.74)	0.67	522 (28.78)	84 (30.77)	0.52	526 (38.20)	127 (30.90)	0.01
Physical activity (*n*%)	324 (12.16)	86 (14.96)	0.07	178 (11.81)	38 (16.67)	0.04	146 (12.61)	48 (13.83)	0.58
**Medical history (*****n*****%)**									
Hypertension (*n*%)	718 (22.50)	250 (36.55)	<0.001	318 (17.53)	71 (26.01)	0.001	400 (29.05)	179 (43.55)	<0.001
Diabetes (*n*%)	216 (6.77)	73 (10.67)	0.0007	98 (5.40)	20 (7.33)	0.21	118 (8.57)	53 (12.90)	0.01
Hyperlipidemia (*n*%)	354 (11.09)	124 (18.13)	<0.001	150 (8.27)	41 (15.02)	0.0007	204 (14.81)	83 (20.19)	0.01
Lipid-lowering drugs (*n*%)	48 (1.50)	13 (1.90)	0.50	25 (1.38)	3 (1.10)	1.00	23 (1.67)	10 (2.43)	0.30
Total stroke (IS, ICH, SAH) (*n*%)	15 (0.56)	9 (1.57)	0.03	8 (0.53)	4 (1.75)	0.06	7 (0.60)	5 (1.44)	0.16
SBP (median, mmHg)	129.33 (116.67, 140.00)	130.67 (120.00, 149.33)	<0.001	121.33 (110.67, 138.00)	130.00 (116.67, 141.33)	0.0002	130.00 (120.00, 143.33)	138.00 (124.67, 150.00)	<0.001
LDL (median, mmol/L)	2.60 (2.18, 3.02)	2.66 (2.06, 3.11)	0.92	2.52 (2.14,2.98)	2.55 (1.94, 3.01)	0.17	2.67 (2.23, 3.09)	2.71 (2.15, 3.15)	0.83
HDL (median, mmol/L)	1.58 (1.32, 1.92)	1.50 (1.28, 1.78)	<0.001	1.67 (1.37, 2.00)	1.61 (1.39, 1.86)	0.01	1.50 (1.26, 1.79)	1.41 (1.21, 1.68)	0.001
TG (median, mmol/L)	1.25 (0.91, 1.84)	1.54 (1.06, 2.21)	<0.001	1.12 (0.82, 1.55)	1.33 (0.94, 1.85)	<0.001	1.49 (1.06, 2.30)	1.73 (1.16, 2.45)	0.002
TC (median, mmol/L)	4.91 (4.34, 5.59)	5.15 (4.55, 5.78)	<0.001	4.89 (4.34, 5.51)	5.14 (4.50, 5.70)	0.0009	4.97 (4.34, 5.66)	5.17 (4.62, 5.86)	0.0003
FPG (median, mmol/L)	5.15 (4.79, 5.69)	5.38 (4.95, 6.09)	<0.001	5.10 (4.73, 5.58)	5.20 (4.86, 5.80)	0.003	5.25 (4.85, 5.82)	5.55 (5.01, 6.44)	<0.001

*BMI, body mass index; FPG, fasting plasma glucose; HDL, high-density lipoprotein cholesterol; hs-CRP, high-sensitivity C reactive protein; LDL, low-density lipoprotein cholesterol; SBP, systolic blood pressure; TC, total cholesterol; TG, triglycerides*.

Results of the comparison of cognitive impairment between each group are shown in Table 3. In all participants, subjects with elevated hs-CRP levels had a lower MMSE score, especially in attention and calculation as well as recall items. In the overweight group, subjects with elevated hs-CRP levels showed the same trends. In the normal weight group, those with cognitive impairment accounted for a larger proportion of participants with elevated levels of hs-CRP and had a lower MMSE score, especially in the recall item.

**Table 3:** Baseline characteristics of MMSE in participants stratified by hs-CRP levels.

**Characteristic**	**Hs-CRP < 3 mg/L**	**Hs-CRP ≥ 3 mg/L**	***P-value***	**Hs-CRP < 3 mg/L, BMI < 25 kg/m^**2**^**	**Hs-CRP ≥ 3 mg/L, BMI < 25 kg/m^**2**^**	***P-value***	**Hs-CRP < 3 mg/L, BMI ≥ 25 kg/m^**2**^**	**Hs-CRP ≥ 3 mg/L, BMI ≥ 25 kg/m^**2**^**	***P-value***
MMSE <24 points	153 (4.79)	43 (6.29)	0.12	77 (4.24)	23 (8.42)	0.01	76 (5.52)	20 (4.87)	0.71
**MMSE item**									
Total	29.00 (27.00, 30.00)	28.00 (27.00, 30.00)	<0.001	29.00 (27.00, 30.00)	29.00 (27.00, 30.00)	0.01	29.00 (27.00, 30.00)	28.00 (27.00, 30.00)	<0.001
Orientation	10.00 (10.00, 10.00)	10.00 (10.00, 10.00)	0.24	10.00 (10.00, 10.00)	10.00 (10.00, 10.00)	0.41	10.00 (10.00, 10.00)	10.00 (10.00, 10.00)	0.41
Registration	3.00 (3.00, 3.00)	3.00 (3.00, 3.00)	0.10	3.00 (3.00, 3.00)	3.00 (3.00, 3.00)	0.17	3.00 (3.00, 3.00)	3.00 (3.00, 3.00)	0.41
Attention and calculation	5.00 (4.00, 5.00)	5.00 (4.00, 5.00)	0.0005	5.00 (4.00, 5.00)	5.00 (4.00, 5.00)	0.16	5.00 (4.00, 5.00)	5.00 (3.00, 5.00)	0.001
Recall	3.00 (2.00, 3.00)	2.00 (2.00, 3.00)	<0.001	3.00 (2.00, 3.00)	3.00 (2.00, 3.00)	0.001	3.00 (2.00, 3.00)	2.00 (2.00, 3.00)	<0.001
Language	9.00 (9.00, 9.00)	9.00 (8.50, 9.00)	0.07	9.00 (9.00, 9.00)	9.00 (8.00, 9.00)	0.06	9.00 (9.00, 9.00)	9.00 (9.00, 9.00)	0.63

*BMI, body mass index; hs-CRP, high-sensitivity C reactive protein; MMSE, Mini-Mental State Examination*.

### Interaction of Hs-CRP Levels With BMI on Cognitive Impairment

Regardless of the hs-CRP stratification, higher BMI was not associated with cognitive impairment. When stratified by age, sex, and education, BMI was also not associated with cognitive impairment. Before and after adjusting for confounders, elevated hs-CRP levels increased the odds of cognitive impairment in normal weight participants (crude OR: 2.08, 95%CI: 1.28–3.37, *p* = 0.003; adjusted OR: 2.06, 95%CI: 1.03–4.10, *p* = 0.04) but not in overweight participants (Hs-CRP <3 mg/L: crude OR: 1.32, 95%CI: 0.95–1.82, *p* = 0.10; adjusted OR: 1.16, 95%CI: 0.76–1.76, *p* = 0.49; Hs-CRP ≥ 3 mg/L: crude OR: 1.15, 95%CI: 0.70–1.91, *p* = 0.58; adjusted OR: 0.74, 95%CI: 0.36–1.52, *p* = 0.89). When additionally stratified by age, sex, and education, no significant interactions between hs-CRP and BMI on cognitive impairment were observed (Table 4).

**Table 4:** Multivariable adjusted odd ratios (OR) for cognitive impairment according to hs-CRP levels and BMI.

	**BMI < 25 kg/m^**2**^**	**BMI ≥ 25 kg/m^**2**^**	**Interaction *P***	**BMI < 2 kg/m^**2**^ & Hs-CRP < 3 mg/L**	**BMI < 25 kg/m^**2**^ & Hs-CRP ≥ 3 mg/L**	**BMI ≥ 2 kg/m^**2**^ & Hs-CRP < 3 mg/L**	**BMI ≥ 25 kg/m^**2**^ & Hs-CRP ≥ 3 mg/L**
Crude OR, 95% CI	1	1.13 (0.85, 1.50)		1	2.08 (1.28, 3.37)^*^	1.32 (0.95, 1.82)	1.15 (0.70, 1.91)
Adjusted OR, 95% CI	1	0.90 (0.62, 1.30)		1	2.06 (1.03, 4.10)^*^	1.16 (0.76, 1.76)	0.74 (0.36, 1.52)
**Sex**			0.21				
Female	1	1.44 (0.74, 2.80)		1	5.52 (1.06, 28.64)^*^	2.09 (1.00, 4.35)^*^	1.53 (0.30, 7.87)
Male	1	0.77 (0.47, 1.25)		1	1.81 (0.79, 4.13)	0.94 (0.55, 1.62)	0.69 (0.27, 1.73)
**Age**			0.65				
<65 years	1	0.86 (0.53, 1.39)		1	2.19 (0.83, 5.79)	1.03 (0.61, 1.76)	0.81 (0.31,2.12)
≥65 years	1	0.97 (0.52, 1.83)		1	2.70 (0.98, 7.42)	1.55 (0.74, 3.26)	0.76 (0.24, 2.43)
**Education, n(%)**			0.72				
Illiteracy or primary school	1	0.74 (0.41, 1.34)		1	2.23 (0.75, 6.64)	0.83 (0.42,1.64)	1.07 (0.37, 3.12)
Middle School	1	1.01 (0.57, 1.80)		1	1.47 (0.48, 4.54)	1.32 (0.71, 2.48)	0.50 (0.14,1.76)
High school or higher	1	1.32 (0.45, 3.89)		1	5.37 (0.84, 34.40)	2.50 (0.72, 8.64)	0.70 (0.06, 8.60)

*BMI, body mass index; hs-CRP, high-sensitivity C reactive protein; MMSE, Mini-Mental State Examination*.

*Adjusted for age, sex, BMI, education, current smoking, drinking, physical activity, hypertension, hyperlipidemia, diabetes, hs-CRP*.

* *P < 0.05*.

When adjusting for confounders to evaluate the association between hs-CRP, BMI, and cognitive impairment sub-items, we found that although elevated hs-CRP level had an association with attention and calculation as well as recall in overweight participants, the interaction of hs-CRP and BMI was not significant, i.e., there was no statistically significant evidence for the interaction between hs-CRP and BMI on any cognitive sub-item (Table 5).

**Table 5:** Multivariate-adjusted odd ratios (OR) for cognitive impairment sub-items according to hs-CRP levels and BMI.

	**BMI < 25 kg/m**^**2**^			**BMI ≥ 2 kg/m**^**2**^			
**MMSE sub-items**	**Hs-CRP <3 mg/L**	**Hs-CRP ≥ 3 mg/L**		**Hs-CRP <3 mg/L**	**Hs-CRP ≥ 3 mg/L**		**Interaction *P***
		**OR, 95%CI**	***P-value***		**OR, 95%CI**	***P-value***	
Orientation	1	1.14 (0.62, 2.09)	0.67	1	1.15 (0.67, 1.99)	0.62	0.90
Registration	1	1.47 (0.67, 3.26)	0.34	1	1.03 (0.52, 2.02)	0.94	0.60
Attention and calculation	1	1.06 (0.79, 1.44)	0.68	1	1.38 (1.07, 1.79)	0.01	0.24
Recall	1	1.28 (0.95, 1.72)	0.10	1	1.53 (1.17, 1.99)	0.001	0.36
Language	1	1.18 (0.83, 1.67)	0.36	1	0.98 (0.72, 1.33)	0.91	0.46

*BMI, body mass index; hs-CRP, high-sensitivity C reactive protein; MMSE, Mini-Mental State Examination*.

*Adjusted for age, sex, BMI, education, current smoking, drinking, physical activity, hypertension, hyperlipidemia, diabetes, hs-CRP*.

## Discussion

In this analysis, we found out the association of hs-CRP in cognitive impairment only in normal weight subjects, but not in overweight subjects. With respect to each MMSE sub-item, elevated hs-CRP had no effect on either overweight or normal weight participants.

According to the World Health Organization (WHO), worldwide, more than 1.9 billion adults (39% of adults) were estimated to be overweight or obese in 2016, of which, nearly one-third were obese (22). By 2030, estimates forecast >50% (3.3 billion people) of the world's adult population will be overweight or obese (23, 24). Overweight status is fast becoming an important clinical and public health burden worldwide. As such, comorbidities related to being overweight are expected to become a public health problem in the coming years. The common comorbidities not only pose health-threatening conditions such as metabolic syndrome, endocrine disorders, cardiovascular diseases, and cancers but also have effects on patients' cognitive functions (25).

Most of the previous studies have shown that the association of obesity and cognitive impairment changes with age. In younger and middle aged adults, overweight and obesity are associated with cognitive impairment, while in old aged people, higher BMIs are associated with better cognition (5–7, 26, 27). A recent meta-analysis showed that in mid-aged people, obesity conferred 1.31-fold excess risk for cognitive impairment, while in old aged people, overweight and obesity conferred a 21 and 25% reduced risk, respectively (5). In our study, there was no significant correlation between obesity and cognition. This may be because the participants in our study were only divided into normal weight and overweight, while previous studies have shown that in midlife, underweight also increases the risk of cognitive impairment as overweight (5), and these participants were classified into normal weight, which may affect our results. In old aged people, a previous Chinese study showed that abdominal obesity might increase the risk of cognitive impairment (28). In China, abdominal obesity is a typical characteristic of the body fat distribution, and most of these people are defined as overweight, which may affect our results.

BMI is typically used to define overweight and obesity, although sometimes by waist-to-hip ratio and waist circumference or fat mass. Moreover, fewer studies have specifically examined the association of BMI and cognitive impairment subtypes. A recent review demonstrated that being overweight, independent of metabolic dysfunction, impairs cognition with respect to executive function, memory, and medial temporal lobe structures (29). Another review found that decision-making, planning, and problem solving were associated with being overweight, but verbal fluency, learning, and memory functions were less affected by obesity (30). A systematic literature review conducted by Prickett et al. to compare evidence for specific cognitive domain deficits showed that nearly all cognitive domains (i.e., attention, memory, verbal fluency, and decision-making) in overweight subjects were impaired; however, the study also showed that as a result of various methodological limitations, the evidence about cognitive domains and obesity was insufficient (31). Further research with same key methods is needed.

There are various pathophysiological mechanisms underpinning overweight status and their impact on cognitive impairment, such as gut microbiota (32), peripheral and central inflammation (15), blood-brain barrier integrity, oxidative stress, and alterations in brain structure or white matter (33, 34). Among these mechanisms, inflammation is commonly conducted over the past years. First, being overweight is associated with low-grade inflammation, and some pro-inflammation cytokines produced by adipose tissues may affect cognition (35). Second, some circulating pro-inflammatory markers such as IL-6 and CRP will likely increase in these subjects and result in global cognitive decline and hippocampal atrophy (36–39). Third, overweight status will aggravate the central tissue injury in the central nervous system by decreasing the gene expression of IL-10 and increasing gene expression of nitric oxide synthase-2 in the human frontal cortex (40). Some animal studies have demonstrated that pro-inflammation in the hippocampus and amygdala may result in overweight-associated cognitive impairment (41). In our study, we found that in the normal weight participants, elevated hs-CRP is associated with cognitive impairment, but not in overweight subjects. This may be because in overweight or obese participants, a variety of mechanisms affect cognition jointly, of which inflammation only play a part role. Moreover, in overweight or obese participants, the inflammation may be more common than in the normal weight (in our study, the median hs-CRP in the overweight and normal groups were 1.30 and 0.80, respectively, *P* < 0.01), which has an impact on cognition for most participants. While in the normal weight participants, the inflammatory factor hs-CRP, as the main factor affecting cognition, may affect cognition with the level increasing through the mechanism above.

Our study has a few limitations. First, some studies showed that being underweight also increased the odds of cognitive impairment, but we did not distinguish between underweight and normal weight in our study (42, 43). Second, compared to the White populations, Asian populations are prone to abdominal fat deposition, which makes the latter have lower BMI but more susceptible to develop metabolic syndromes (44) that also affect cognition (45). Third, the criteria for measuring overweight and cognition impairment were not uniform, and the most commonly used measurements scales, i.e., BMI and MMSE were applied in our study. Fourth, hs-CRP and BMI was collected at baseline in 2010 and the MMSE was examined during follow-up visits in 2012, which may not exactly reflect the participants' cognition. However, since we selected healthy adults without stroke and other major diseases as well as cognitive impairment, the follow-up MMSE can represent cognitive status. Fifth, this cohort was a subgroup from the APAC study, so the findings might not be generalizable to other populations. Advanced studies are essential for further research.

## Conclusion

Our study suggests that elevated hs-CRP levels increase the odds of cognitive impairment in normal weight participants, but not in overweight participants. Elevated hs-CRP levels have no effect on any cognitive sub-item.

## Data Availability Statement

The raw data supporting the conclusions of this article will be made available by the authors, without undue reservation.

## Ethics Statement

The APAC study was approved by the Ethics Committees of the Kailuan General Hospital and Beijing Tiantan Hospital. The patients/participants provided their written informed consent to participate in this study.

## Author Contributions

XZ: study concept and design. JW: drafted the manuscript. AW: statistical analysis. All authors contributed to the article and approved the submitted version.

## Conflict of Interest

The authors declare that the research was conducted in the absence of any commercial or financial relationships that could be construed as a potential conflict of interest.
